# Cincinnati Letter

**Published:** 1883-12

**Authors:** 

**Affiliations:** Cincinnati


ARTICLE IV.

CINCINNATI, NOV. 6, 1883.

MR. EDITOR:

Cincinnati, the Queen of the West, the City of Music, the city containing more Germans to the square mile than any other spot outside of the Fatherland, and more dirt than any other city outside of Pittsburgh; the city of Graham, Drake, Blackburn, Longworth, the Musseys—illustrious father and devoted son—and Bartholow, is sometimes thought to be jealous of Chicago; but she is not. She looks with pride and wonder upon her overgrown younger sister, remembering well when one of her solid citizens could have bought the village on the lake.

Cincinnati has, according to the directory for 1883, 463 doctors, regular, irregular, and defective. Many of these are practicing pharmacy, others dentistry, while some have the title having taken it as a matter of education, while many are going through that trying ordeal, sitting around waiting for a practice, and others, having worried through, are simply waiting to die. The number of active practitioners, in proportion to the population, is comparatively small. It must be considered, however, that the large proportion of the midwifery work here is done by midwives, while old women and many other forms of the vulture tribe, prey on the poor practitioner.

Cincinnati has five hospitals. First, and largest, is the Cincinnati. A better building for a hospital, all things considered, the writer has not seen either in this country or Europe, unless, perhaps, St. Thomas' Hospital in London. The staff consists of members of the Faculties of the Medical College of Ohio and of the Miami Medical College, and a number not belonging to these colleges. Its clinics are open to all students alike. It is supported by the city. Second in size, and number of its patients, is the St. Mary's Hospital. This is patronized largely by the Germans, probably 80 per cent. of the patients being of that class. It is under the charge of the Sisters of the Poor of St. Francis, and is supported by charity. Its staff consists, for the most part, of men not connected with a college but the Medical College of Ohio is represented. Third, is the Hospital of the Good Samaritan under the entire control of the Faculty of the Medical College of Ohio, its students only being admitted to the clinical lectures. Next comes the U. S. Marine Hospital, and then the Jewish Hospital. Besides, there are half a dozen private hospitals which to mention would be but to advertise.

Cincinnati has two medical colleges—the Medical College of Ohio, established in 1819, and having an average attendance of about 330 students; the Miami Medical College, the result of an un-united fracture of the Faculty of the Medical College of Ohio, which has resisted the efforts of both conservative and radical surgery. The students in attendance average about 110. There are other corporations, 'tis true, but they are irregular and defective, and it is bad enough to have them forced upon one's thoughts occasionally, without mentioning them. Besides these, we have one of the oldest dental colleges in the world, and a school of pharmacy. The Cincinnati University last summer made overtures to the College of Pharmacy proposing to absorb it, but the College of Pharmacy did not prove that kind of cotton. The University has made like overtures to the medical colleges, and met with like responses.

Cincinnati has two medical libraries; that in the Public Library being chiefly devoted to books, having the best of the American, English, French and German journals. That in the Cincinnati Hospital, though deficient in books, is rich in the journals of America and Europe.

Cincinnati has five medical societies. The Hippocratian, consisting of the ex-internes at the Cincinnati Hospital, meets annually.

The Obstetrical Society is a select body of men limited to twenty. Their meetings are "catamenial," they are a "regular" body, they "labor" hard, and the "flow" of wisdom is great. It is needless to remark that those members who have not reached the menopause are nearing it. Hæmatoma of the Vulva was one of the interesting cases reported at the last meeting by Dr. W. H. Wenning.

The Drake Medical Society, organized only last spring, is the youngest medical society in the city. It is also select. Its members number but fifteen, are the young members of the profession, and are all graduates of the Medical College of Ohio, and were in college at the same time. They meet at each others' offices, and are economical; they have to be. Interesting papers at recent meetings were: "The Use of Opium in the Diseases of Children," Louis Schwab, M.D.; "Otitis Media Purulenta," Chas. W. Tangeman, M.D.; "Gonorrhœa," W. H. Kelley, M.D.; "Ovariotomy," Chas. T. Locke, M.D. E. W. Mitchell, M.D., President; Geo. A. Fackler, M.D., Secretary. Meets every Tuesday evening.

The Cincinnati Medical Society, a fragment of the previously mentioned un-united fracture, meets every Tuesday night. Recent interesting papers were: "Scurvy," J. E. Eichberg, M.D.; "Locomotor Ataxia," Philip Zenner, M.D.; "Scarlatinal Rheumatism," W. H. Taylor, M.D. B. P. Goode, M.D., President; W. H. McReynolds, M.D., Secretary.

Cincinnati Academy of Medicine, established 1857, meets every Monday night. Interesting papers have been read recently on: "Color Blindness," Julia W. Carpenter, M.D.; "Cephalhæmatoma," with report of a case of the intra-cranial variety, E. S. McKee, M.D.; "The Despotism of Words in Science," Orpheus Everts, M.D. Cases of hæmorrhage from the bowels were reported by J. T. Whittaker, M.D.; cases pertaining to surgical treatment of pleuritic effusions were reported by Drs. Ransohoff, Whittaker, Jones, Krause and Mitchell; a case of "Abdominal Tumor," by James T. Whittaker, M.D., and Joseph Ransohoff, M.D. W. W. Seely, M.D., Dean of the Medical College of Ohio, is President, and W. H. Wenning, M.D., of the St.

Mary's Hospital, is Secretary. Short-hand reports are taken in full and printed.

Cincinnati has three medical journals—one weekly and one monthly, both devoted to general medicine and surgery; the other, a monthly, devoted to obstetrics and diseases of women and children.

The health of Cincinnati during the past summer and autumn has been marvelous. The week ending October 27, 1883, reported only 68 deaths, or 12.66 per 1,000, the lowest for ten years. The weekly death-rate has been below 100 all summer. This is bad for the doctors, for there has been a corresponding small amount of sickness, but for the undertakers it has been terrible. The poor fellows have been compelled to add on 50 per cent. to their charges, to keep up appearances.

In June the Legislature abolished the Health Board of Cincinnati, in answer to a petition from her citizens, and ordered an appointment by the judges of the Superior court. This, said judges refused to do, and the city was two months without health board or officer. Common Council then took the matter in hand, and elected another Health Board, which has been serving since August 1. It filled out the appointments, and so far seems to have disappointed everybody by doing its duty. District physicians, so-styled assistant health officers, were appointed, one for each ward, twenty-five in all, at a salary of $25.00 each per month. They are expected to look after the sanitary condition of the ward and doctor the sick poor. The city is entirely free from small-pox, and has been for one month past. Small-pox here makes sad havoc at times in the German quarters, as they number many anti-vaccinationists. The Health Officer has been struggling with the dessicating companies, the so-called "stink" factories, but he always comes off second. Money is a powerful power.

A pusillanimous penny sheet here has for the past two weeks been dosing out the doctors a half dozen each issue. It pretends to give a biographical sketch. Gives the age, height, weight, color of his hair, whiskers, eyes, etc., his manner of dress, of visiting his patients, his habits, and views on tobacco, temperance and religion. His residence and office, place of graduation, how long in practice and how much his practice was worth. As to the latter point many were the untruths told; doctors whose practice hardly paid their office rent were down for $7,000 per annum, while another who is known to make visits for fifty cents was put down for $15,000 to $18,000. Every article had some attempt at wit, such as "a strawberry blonde," "whiskers of a crushed strawberry hue," "somewhat of a dude in his dress," etc., etc. Of course the whole thing was disgusting. Some doctors were written up, from no fault of theirs, the reporter having either obtained his points from other parties or from observation and imagination; others, of course, pushed their way in and were happy over a little cheap notoriety. The whole matter goes to show the greatest failing of the medical profession, viz. to exaggerate the amount of their income. It creates wrong impressions among the laity, causing them to think every doctor rolling in wealth, which is not the case. Then, too, it has the effect of making bills harder to collect, and every one knows now they are difficult enough.

Sir William MacCormac, I see, has been at Chicago, and the defendant at a reception at the Grand Pacific. Sir William visited also St. Louis and Louisville but evaded Cincinnati. Possibly it was because Cincinnati's great lights were not persistent enough in their inducements. Sir William is an elegant fellow and deserves great praise for the exceptional manner in which he conducted the International Medical Congress of '81; possibly he deserves the, in England, great honor of the Knighthood, but, we think, not half so much as that indefatigable and life-long worker in the profession, the unassuming, in England unexcelled, Johnathan Hutchinson. Who of the profession haven't for years heard the name of Johnathan Hutchinson on the lips of their brethren and seen it cut in the teeth of their patients? Who ever heard of Sir William MacCormac before the Congress of '81?

There has, for two months past, been a controversy going on in Cincinnati between the physicians and druggists. Complaints were made first in the Academy of Medicine early in September that many of the druggists were prescribing, that they were treating more gonorrhœa than the physicians; that they displayed too prominently the advertisements of quacks and their nostrums; that they in many instances sent medicine, ordered by physicians, wrapped up in quack medicine advertisements; that they substituted; that they refilled prescriptions too freely; that they advised patients of one doctor to discontinue him, recommending another; that in one instance one spoke disparagingly of medicine ordered by a physician, saying he had "a clapp mixture which beat that all to pieces;" that two were advertising their gonorrhœa mixtures in the daily papers, etc., etc.

A committee was appointed to confer with like committees from the Cincinnati Medical Society and the College of Pharmacy.

The druggists filed counter claims that the physicians were in some instances dispensing their own medicine and that they were prescribing proprietary and secret remedies.

After considerable discussion among the committees the following rules have been drawn up:

I. That pharmacists shall not prescribe medicines.

II. Pharmacists shall not too frequently fill prescriptions which might lead to disease or vicious habits.

III. Pharmacists shall only supply medicines on prescriptions, or where the complaint is stated by the customer to be one dangerous to life.

IV. The right to refill the prescriptions when presented by the person to whom they were issued by the physician is acknowledged.

V. Physicians should not dispense medicines except in cases of emergency, nor prescribe proprietary, secret or copyrighted medicines.

VI. The prescription when in the hands of the pharmacist and filled, shall be the latter's property, as they may be necessary for protection.

VII. The use of prescription blanks by physicians with the address of any individual pharmacist on them is liable to misconstruction and shall be discontinued.

VIII. The evils arising from the wholesale use of patent medicines is recognized and their use discountenanced.

IX. A Board of Arbitration, consisting of five physicians and five druggists, shall be appointed, who shall subscribe to these rules. This Board shall hear all complaints against any subscriber to these rules for their violation and shall decide the disputes.

The disagreeable part of this is that the daily press has been writing us up for several days and making more out of it than any one else. Respectfully,

ABRACADABRA.

---

MOLLUSCUM SIMPLEX IN THE LABIUM MAJUS.

A girl perfectly healthy, twenty-one years of age, noticed a swelling on the genital parts two years ago, but since eight months the tumor grew rapidly. Three weeks ago while rubbing the tumor the latter burst and there was a great deal of hæmorrhage. The tumor is pedunculated and the pedicle is inserted on the labium majus of the left side. The insertion is linear, and extends on the whole length of the labium majus. Its height is that of the lip and its external surface corresponds with that of the labium majus. The external appearance of the pedicle is exactly that of the labia. The body of the tumor consists of a certain number of lobules some being large, others adjoining these being small, and there are yet others which are placed like parasites on the other. The whole form resembles a grape. The epithelium of all the lobules is that of the genital parts, but there is between the lobes an erosion from which flows a bloody liquid. The lobes are in the form of a sac and resemble false hæmorrhoides. The removal of the tumor was executed without loss of blood.—*Le Progrés Médical.*

